# 

**Published:** 1888-09-01

**Authors:** 


### "The Hospital" Library of New and Standard Works.

For all information respecting the appearance of Titles, Prices, etc., of Books in this page, apply to Mr. W. H. Howe, office of The Hospital, 38, Old Jewry.

Crown 8vo., cloth, 2s. 6d.

THE COMMONER DISEASES AND ACCIDENTS TO LIFE AND LIMB. Firstly, their Prevention; secondly, their Immediate Treatment. By M. M. BASIL, M.A., M.B., C.M. (Edin.).

"Brief, clear, and good."—*Athenæum.*

"Thoroughly readable."—*Hospital.*

London: J. and A. Churchill, 11, New Burlington Street.

11th edition, 2s, post free for 26 stamps.

STAMMERING, STUTTERING, LISPING, &c.; their Causes and Cure. By W. ABBOTTS, M.D., Physician to the Hospital for Speech Affections.

Beaumont and Co., 6, Wells Street, Oxford Street, W. (72)

### To Residents, Secretaries, and Matrons.

The Editor will be glad to have the Regulations and each Report as issued by Asylums, Hospitals, Convalescent and Nursing Institutions, as well as those of other Charities, and an early copy in duplicate of all Appeals and Festival Notices, etc., addressed to *The Editor, The Lodge, Porchester Square, London, W.* Any superintendent, secretary, matron, or other chief official who regularly sends such information may be registered, on application to the Publisher, to receive free of cost a cover for binding The Hospital in half-yearly volumes.

### Notice to Advertisers and Subscribers.

All Advertisements and Business Communications should be addressed to The Publisher, *not to the Editor*, The Hospital, 38, Old Jewry, E.C. A Scale of Charges will be found in the Advertisement Sheets, page iii.

### THE STUDENT'S COLUMN.

### SOCIETY OF APOTHECARIES OF LONDON.

THE following gentlemen have been appointed assistant examiners to the society by the General Medical Council, and conduct the examinations in surgery and anatomy:—*Andrew Clark, F.R.C.S.; *W. Adams Frost, F.R.C.S.; *W. Arbuthnot Lane, F.R.C.S.; *G. H. Makins, F.R.C.S.; *W. J. Walsham, F.R.C.S. The following gentlemen have been appointed by the society as examiners for the ensuing year:—*Henry Bullock, F.R.C.S.; H. Radcliffe Crocker, M.D., B.S., Lond., F.R.C.P.; *William Duncan, M.D., M.R.C.P., Lond.; *F. de Havilland Hall, M.D., Lond., F.R.C.P.; *Robert James Lee, M.D., Cantab., F.R.C.P.; *A. H. N. Lewers, M.D., Lond., M.R.C.P.; William Robert Smith, M.D., D.Sc., F.R.S. Ed.; *John Sherwood Stocker, M.D., Lond., M.R.C.P. (chairman of the Court of Examiners); *John Charles Thorowgood, M.D., Lond., F.R.C.P.; Francis Warner, M.D., Lond., F.R.C.P. Dr. Klein, F.R.S., has been appointed the society's examiner in physiology for the ensuing year. Dr. Charles Alfred Hebbert has been appointed assistant examiner by the society for the ensuing year.

*Members of the Court of Examiners.

### PASS LIST.

The following having satisfied the Court of Examiners during the past month (June) as to their knowledge of the science and practice of medicine, surgery, and midwifery, received certificates entitling them to practise as licentiates of the society:

*Best, William Harris*, London Hospital.
*Harris, Percival Seymour*, Middlesex Hospital.
*Hulbert, Henry Harper*, Oxford, and St. Thomas's, London.
*Hill, Robert*, Middlesex Hospital.
*Motte, William de la*, London Hospital.
*Midelton, William John*, St. Bartholomew's Hospital.
*Sargent, William Gostwycke*, London Hospital.
*Smith, John Arthur*, Leeds.
*Stovin, Cornelius Frederick*, London Hospital.
*Sheldon, Robert Garnett*, Victoria University, Liverpool.
*Stephenson, Owen Taunton*, University College, Liverpool.
*Sleeman, Richard Reginald*, Cambridge, and St. Mary's, London.
*Webb, Helen*, Royal Free Hospital
*Whitaker, George Herbert*, St. Bartholomew's Hospital.

The following passed in surgery:

*Bradley, G. M.*, Grant Medical College.
*Burgess, J. G.*, Guy's Hospital.
*Cornilliac, J.*, King's College.
*Gilpin, R. H*, Middlesex Hospital.
*Gunn, F. W.*, King's College.
*Lory, A. G. B.*, London Hospital.
*Lange, A P*, King's College.
*Mitchell, E. S.* Queen's University, Kingston.
*Wilson, A. R.*, Oxford University.

The following passed in medicine:

*Holt, H. M.*, Leeds.

At the examination in arts, of 217 present, 2 passed in the first class, 30 in the second class, and 148 passed in one or more subjects, but not in all.

The following are the questions given at the July Examinations:—

#### EXAMINATION IN MEDICINE.

MEDICINE.—Four questions only to be answered.

1. Give the various causes of dysphagia so far as they affect the œsophagus, and describe the treatment you would employ in any two of the forms.
2. Describe a case of German measles, and give the differential diagnosis from ordinary measles.
3. Give the ætiology, pathology, symptoms, and treatment of locomotor ataxy.
4. Describe a case of myxœdema, and state with what diseases it may be confounded.
5. How would you treat convulsions with albumenuria coming on after scarlatina?
6. Describe the varieties of purpura. What is the pathology of this disease? And how should it be treated?

THERAPEUTICS.—One question only to be answered.

1. Give the dietetic and medicinal treatment of the uric acid diathesis.
2. What remedies are used for the relief of pain and spasm? And give the indications for their employment.

PATHOLOGY.—Two questions only to be answered.

1. Describe the pathology of rickets.
2. Give the pathology of lymphadenoma.
3. Give an account of the morbid changes to which arteries are liable.

FORENSIC MEDICINE AND HYGIENE.—Three questions only to be answered.

1. What means have been adopted for the induction of criminal abortion? And what evidences of it may be found *post mortem*?
2. Describe a case of general paralysis of the insane.
3. What are the symptoms of poisoning by carbolic acid, and what is the appropriate treatment?
4. How would you detect corrosive sublimate and sugar of lead, respectively, in the contents of the stomach?

### EXAMINATION IN OBSTETRIC MEDICINE.

Midwifery.—Three questions only to be answered.

1. How would you proceed to effect delivery at full term in a patient whose pelvis had the following measurements: Between the anterior superior iliac spines, $10\frac{1}{2}$ inches; maximum distance between iliac crests $10\frac{1}{4}$ inches; external conjugate, $5\frac{3}{4}$ inches; inclined (or diagonal) conjugate, $3\frac{3}{8}$ inches? *Note.—The Vertex is supposed to be presenting.*

2. Describe the mechanism of labour in a face presentation, where the chin is behind and to the left.

3. Describe fully the operation known as podalic version, and mention the cases in which you would perform it.

4. What measures would you adopt for the treatment (prophylactic and curative) of post partum hæmorrhage?

Gynæcology.—One question only to be answered.

1. Give the symptoms and signs met with in the case of an ordinary multilocular ovarian cyst. How would you distinguish between it and a uterine fibroid?

2. Discuss the etiology and treatment of dysmenorrhœa.

### EXAMINATION IN SURGERY.

Surgery.—Four questions only to be answered.

1. In a case of scalp-wound, what would lead you to suspect inter-cranial suppuration? How would you deal with such a condition?

2. Give the causes, symptoms, treatment, and prognosis of extravasation of urine.

3. What are the symptoms of aneurism of the common femoral artery For what may it be mistaken? Describe the treatment you would adopt.

4. Give the signs, diagnosis, treatment, and prognosis of soft chancre.

5. Enumerate the conditions for which excision of the eyeball may be required. Give a detailed description of the operation.

Surgical Pathology.—One question only to be answered.

1. Give a brief account of the pathology of the various forms of goître.

2. Describe the changes that take place in the urinary apparatus in a case of neglected stricture.

Surgical Anatomy.—One question only to be answered.

1. Describe the inguinal canal, and give the coverings of direct and indirect inguinal hernia.

2. Give the origin, insertion, and nerve-supply of the muscles at fault in talipes equino-varus.

---

### AMUSEMENTS WITH PROFIT FOR ALL AGES.

**Rules.**—All answers must be addressed to the Prize Editor, The Hospital, 38, Old Jewry, Cheapside, London, E.C., not later than the *first* post on Thursday next. The full name and address must in all cases be given, but a *nom de plume* may be added for publication. Only one side of the paper must be written on.

### FOR MEN AND WOMEN.

**Rule.**—Competitors can enter for *all* quarterly competitions, but no competitor may take more than *two quarterly prizes* during the year.

### First Quarterly Poetical Competition.

Commenced May 26th, 1888; ended August 25th, 1888.

This quarter a PRIZE of ONE GUINEA will be given to the competitor who gains the highest number of marks for original poems, on any subjects suitable for insertion in this paper. All contributions sent in will be credited according to merit, eighteen marks being the highest score for any one contribution. This competition to alternate weekly with the set acrostic competition.

### Acrostic Competition.

This quarter the original acrostic competition will be discontinued, and A PRIZE of HALF-A-GUINEA will be given to the competitor who gains the highest score for solution of acrostics set. *One mark* only will be given to the competitors who solve all the lights of each acrostic.

Exception will be made in the case of *quotation acrostics*, when *two marks* will be given, *one* for the correct solution of all *lights*, and *one* mark for the *source* of the quotations.

### Answers.

No. 6.—Double Acrostic.

D elph I
Y ar N
S el F
P everi L
E m U
P eige E
S eda N
I ne Z
A bracadabr A

Many answers have been received, but Reldas only has succeeded in guessing every light correctly.

We shall publish results of Acrostic and Poetical Competitions next week.

### SPECIAL NOTICE.

The Competitions in this column will be suspended until the autumn, when a new scheme will be presented, in which we hope that all our present competitors, and many new ones, will take part.

### THE CHILDREN'S CORNER.

PRIZES.

FOR MOST CORRECT ANSWERS TO PUZZLES.

This Commenced October 1st, 1887.

One Pound a Month.

A PRIZE OF THREE GUINEAS

to the Competitor who shall have sent in the largest number of correct or best answers during the twelve months.

### Puzzles—First Week.

No. 1. Geographical Enigma.—I am the name of a leather, and also a State in Africa.

No. 2. Charade.

My *first* is either bad or good,
May please or may offend you;
My *second* in a thirsty mood
May very much befriend you.
My *whole*, though termed a "cruel word,"
May yet appear a kind one;
It often may with joy be heard,
With tears may often blind one.

| | | | | | | | | |
|---|---|---|---|---|---|---|---|---|
| | | | | a | | | | |
| | | | a | a | a | | | |
| | | c | c | d | d | e | | |
| | e | e | e | e | e | e | e | |
| g | h | i | i | i | l | l | n | n |
| | n | n | o | o | p | p | p | |
| | | p | p | p | p | r | | |
| | | | r | r | v | | | |
| | | | | y | | | | |

1. A consonant.

2. Noise.

3. Sweet.

4. Sour.

5. A fruit.

6. Cut short.

7. Merry movement.

8. A beverage.

9. A vowel.

No. 4.—What all children are very fond of, and the manner in which it should be done will give you the name of a celebrated mathematician.

### Results of Third Week Puzzles.

No. 9.—Walpole, Pitt, Burke, Fox.

No. 10.—Double Acrostic

R echa B
I c E
G lendowe R
H ahneman N
I ndor E

No. 11. Decapitation.—Wheel, Heel, Eel.

No. 12. Square Word.

E V E R
V E R E
E R N E
R E E L

| Marks. | August 18th. | Total 1st 2nd, and 3rd Weeks. |
|---|---|---|
| A. C. K. | 4 | 12 |
| Alsie | — | 1 |
| Boadicea | 3 | 10 |
| Curly | — | 4 |
| Hercules | — | 2 |
| Poodle | — | 2 |
| Thanet | 3 | 11 |
| Scrooge | 1 | 9 |
| Gem | 3 | 10 |
| Ally | 4 | 11 |
| G. M. M. | 4 | 11 |
| Janette | — | 7 |
| Tynesider | 4 | 10 |
| R. H. | — | 7 |

### Conditions.

I. Every paper sent in must be clearly written, and one side only must be used. All competitors must mark at the head of their papers the number of their competition.

II. Each paper must be signed by the author with his or her real name and address. A *nom de plume* may be added, if the writer does not desire to be referred to by us by his real name. In the case of all prizewinners, however, the real name and address will be published.

III. All communications relating to prize competitions should be addressed to "The Prize Editor, The Hospital, 38, Old Jewry, London, E.C." Competitors are requested not to include in letters, etc., to the Prize Editor communications on matters of other business. All letters must be fully prepaid.

IV. The Prize Editor of The Hospital is the sole judge of the competitions, and his decision is in all cases final and without appeal.

V. The Prize Editor reserves the right to publish any paper sent in, whether it receives a prize or not. He does not hold himself responsible for any MSS. sent, nor can he undertake to return rejected competitions.

VI.—All children resident at school are requested to forward their *home* address in addition to the school one, when entering for the "Children's Corner" Competitions.

**Notice to all Correspondents.**—N.B.—All letters referring to this page, which do not arrive at 38, Old Jewry, Cheapside, London, E.C., by the *first post on Thursdays*, and are not addressed PRIZE EDITOR, will in future be disqualified and disregarded.

No one will be permitted to win the Monthly Prizes twice in any one year, the Annual Prize being offered especially to prevent this.

